# Endotype–phenotyping may predict a treatment response in progressive fibrosing interstitial lung disease

**DOI:** 10.1016/j.ebiom.2019.10.050

**Published:** 2019-11-12

**Authors:** Anna-Maria Hoffmann-Vold, S. Samuel Weigt, Rajan Saggar, Vyacheslav Palchevskiy, Elizabeth R. Volkmann, Lloyd L. Liang, David Ross, Abbas Ardehali, Joseph P. Lynch, John A. Belperio

**Affiliations:** a Department of Rheumatology, Oslo University Hospital, Rikshospitalet, Pb 4950 Nydalen, 0424 Oslo, Norway; b Institute of Clinical Medicine, University of Oslo, Rikshospitalet, Pb 4950 Nydalen, 0424 Oslo, Norway; c Department of Medicine, David Geffen School of Medicine at UCLA, 10833 Le Conte Ave, Los Angeles, CA 90095, USA; d Department of Surgery, UCLA, 10833 Le Conte Ave, Los Angeles, CA 90095, USA

**Keywords:** Interstitial lung disease, Idiopathic pulmonary fibrosis, Connective tissue disease, Proteins, Outcome, ANOVA, analysis of variance, CSF1R, colony stimulating factor 1 receptor, CTD, connective tissue disease, DLCO, diffusing capacity for carbon monoxide, FDA, Food and Drug Administration, FGF, fibroblast growth factor, FGFR, fibroblast growth factor receptor, FVC, forced vital capacity, GAP, Global Alignment and Proportion, HP, hypersensitivity pneumonitis, HRCT, high resolution computed tomography, ILD, interstitial lung disease, IPF, idiopathic pulmonary fibrosis, Lck, lymphocyte-specific tyrosine protein kinase, M-CSF1R, M-colony stimulating factor, PDGF, Platelet derived growth factor, PDGFRα, Platelet derived growth factor receptor-alpha, PDGFRβ, Platelet derived growth factor receptor-beta, PF-ILD, progressive fibrosing-ILD, RA, rheumatoid arthritis, SD, Standard deviation, 6MWD, six minute walking distance, SSc, systemic sclerosis, Tfh, T follicular helper cells, TGF-β, transforming growth factor beta, Th2, T2 helper cells, UCLA, University of California Los Angeles, VEGF, Vascular Endothelial Growth Factor, VEGFR, Vascular Endothelial Growth Factor Receptor

## Abstract

**Background:**

Some interstitial lung disease (ILD) patients develop a progressive fibrosing-ILD phenotype (PF-ILD), with similar persistent lung function decline suggesting common molecular pathways involved. Nintedanib, a tyrosine kinase inhibitor targeting the PDGF, FGF, VEGF and M-CSF pathways, has shown comparable efficacy in idiopathic pulmonary fibrosis (IPF) and systemic sclerosis-associated ILD (SSc-ILD). We hypothesize that Nintedanib targeted molecular pathways will be augmented to a similar degree across PF-ILD regardless of aetiology.

**Methods:**

We collected explanted lung tissue at the time of lung transplantation from 130 PF-ILD patients (99 (76%) IPF, 14 (11%) SSc-ILD, 17 (13%) other PF-ILD), and wedge biopsies from 200 donor lungs and measured PDGF, FGF, VEGF and M-CSF concentrations by Luminex.

**Findings:**

The concentrations of PDGF-AA, PDGF-BB, FGF-2, VEGF and M-CSF were significantly increased in PF-ILD lungs compared to donor lungs (PDGF-AA 93·0 pg/ml [±97·2] vs. 37·5 pg/ml [±35·4], *p* < 0·001; PDGF-BB 102·5 pg/ml [±78·8] vs. 61·9 pg/ml [±47·0], *p* < 0·001; FGF-2 1442·4 pg/ml [±426·6] vs. 1201·7 pg/ml [±535·2], *p* = 0·009; VEGF 40·6 pg/ml [±20·1] vs. 24·9 pg/ml [±29·5], *p* < 0·001; and M-CSF 25526 pg/ml [±24,799] vs. 6120 pg/ml [±7245], *p* < 0·001). There were no significant differences in these growth factor/angiogenic molecules/cytokine concentrations when segregated by IPF, SSc-ILD and other PF-ILDs.

**Interpretation:**

Nintedanib specific targeted molecular pathways are augmented to a similar magnitude in all PF-ILD lung tissue as compared to controls, suggesting that Nintedanib treatment may be efficacious in PF-ILD regardless of aetiology. We speculate that clinical trials using Nintedanib for PF-ILD with or without IPF or SSc-ILD should show a similar relative reduction in FVC decline as seen in IPF and SSc-ILD (∼45–50%).

**Funding:**

Health Grant P01-HL108793 (JAB), South-Eastern Norway Regional Health Authority Grant 2018072 (AMHV).

**Research in context**

**Evidence before this study**

Although the different subsets of interstitial lung disease (ILD) may differ with regard to their initiation/trigger (e.g., unknown for idiopathic pulmonary fibrosis (IPF) and sarcoidosis, autoantigen with adaptive immune responses for connective tissue disease (CTD-ILD), antigen exposure for chronic hypersensitivity pneumonitis (HP), radiation for x-ray induced fibrosis, coal or silicosis or siderosis for pneumoconiosis); many patients develop progressive fibrotic ILD (PF-ILD) with similar clinical courses regardless of ILD aetiology. Nintedanib is an intracellular inhibitor of tyrosine kinase receptors that is predominately involved in PDGFRα, PDGFRβ, FGFR 1–3 and VEGFR 1–3 pathways. In vitro and in vivo studies have suggested that all of these pathways are involved in ILD pathobiology via inflammation/cell activation, fibroplasia and vascular remodeling. To date, it has not been investigated whether the predominant pathways Nintedanib inhibits are collectively augmented in the lungs of patients with PF-ILD.

**Added value of this study**

We demonstrate for the first time that concentrations of Nintedanib-specific targeted proteins are all elevated in explanted lung tissues from patients with PF-ILD, regardless of aetiology. Concentrations of PDGF-AA, PDGF-BB, FGF-2, VEGF and M-CSF in whole lung homogenates from patients with PF-ILD of different aetiologies including IPF, systemic sclerosis (SSc) ILD, other CTD-ILD, sarcoidosis and exposure related-ILD were augmented as compared to healthy donor lungs.

**Implications of all the available evidence**

Collectively, these results suggest that Nintedanib target pathways (PDGF-AA, PDGF-BB, FGF-2, VEGF and M-CSF) are augmented in all PF-ILD subsets and importantly their concentrations are similar across PF-ILD subsets suggesting that Nintedanib treatment should have similar efficacy in PF-ILD regardless of aetiology and should have a relative reduction in the annual rate of decline in FVC (mL/year) of approximately 45–50% for PF-ILD.

**CRediT authorship contribution statement**

**Anna-Maria Hoffmann-Vold:** Conceptualization, Data curation, Formal analysis, Methodology, Writing - original draft, Writing - review & editing. **S. Samuel Weigt:** Conceptualization, Data curation, Formal analysis, Methodology, Writing - original draft, Writing - review & editing. **Rajan Saggar:** Formal analysis, Methodology, Writing - original draft, Writing - review & editing. **Vyacheslav Palchevskiy:** Formal analysis, Methodology, Writing - original draft, Writing - review & editing. **Elizabeth R. Volkmann:** Formal analysis, Methodology, Writing - original draft, Writing - review & editing. **Lloyd L. Liang:** Formal analysis, Methodology, Writing - original draft, Writing - review & editing. **David Ross:** Formal analysis, Methodology, Writing - original draft, Writing - review & editing. **Abbas Ardehali:** Formal analysis, Methodology, Writing - original draft, Writing - review & editing. **Joseph P. Lynch:** Conceptualization, Data curation, Writing - original draft, Writing - review & editing. **John A. Belperio:** Conceptualization, Data curation, Formal analysis, Methodology, Writing - original draft, Writing - review & editing.

Alt-text: Unlabelled Box

## 1 Introduction

Interstitial lung disease (ILD) encompasses a large group of varying parenchymal lung disorders, most of which are classified as rare [1,2]. Lung fibrosis progresses in many patients with ILD, and is associated with impairment in quality of life, declining functional status and early mortality [3,4]. Idiopathic pulmonary fibrosis (IPF), may be regarded as the “prototype” progressive fibrosing ILD (PF-ILD), and is, by definition, characterized by progressive lung fibrosis, progressive lung function decline and worsening of symptoms regardless of treatment [5,6]. Although the presence of progressive fibrosis is characteristic, it is not restricted to IPF [7]. ILD subtypes, which are at risk of developing a progressive fibrosing phenotype include ILDs associated with connective tissue disease (CTD-ILDs) such as rheumatoid arthritis (RA-ILD) and systemic sclerosis (SSc-ILD); sarcoidosis; hypersensitivity pneumonitis (HP); and exposure-related ILDs such as pneumoconiosis. Recently, there has been great interest in the potential value of “lumping” all forms of PF-ILD regardless their aetiology; however identifying mechanisms responsible both for the development and for progression of fibrotic ILDs are important to support this concept [8].

From a pathophysiological standpoint, fibrosis almost universally results when there is irreparable loss of epithelial or, to a lesser degree, endothelial barrier integrity, seen in IPF and in many of the non-IPF ILDs, in particular CTD-ILD and HP [9,10]. Although the aetiology of cellular injury may vary, be it autoimmune mechanisms in CTD-ILD, inhaled organic dust particles in HP, or an unknown injury in IPF, the initiation of the fibrotic response is likely to be related to persistent injury and aberrant repair. While mechanisms of disease initiation may differ from that of progression, contrariwise it is possible progression falls into a more conserved spectrum of pathobiological mechanisms, including growth factor activation, cytokine and chemokine alterations and epigenetic reprogramming of fibroblasts with ongoing vascular remodeling leading to disease progression that eventually causes loss of FVC and mortality in all PF-ILD [10], [11], [12], [13], [14]. From a drug targeting perspective, treatment with Nintedanib has focused on reducing the loss of forced vital capacity (FVC) over time, which is effecting disease progression [15,16]. Consequently, from a disease progression/treatment standpoint it may be more useful to “lump” diverse fibrotic/ILD disorders into one (e.g., PF-ILD) [7,8].

To date, there are no treatment recommendations for ILDs other than IPF and SSc-ILD and only IPF has Food and Drug Administration (FDA) approved treatments with Nintedanib being one of them [5,^6^,[17], [18], [19]. Interestingly, studies have shown that many of the PF-ILD, regardless of the ILD aetiology, have a similar clinical course that consist of ongoing loss of lung function over time [7,20]. Nintedanib is a tyrosine kinase inhibitor that predominately targets the PDGF, FGF, VEGF, and M-CSF pathways [15,16]. Recently, Nintedanib treatment was associated with a relative reduction in the annual rate of decline in FVC (mL/yr) in SSc-ILD of 44%, which is similar to that shown in IPF with 49% [5,21]. This suggests Nintedanib targeted pathways may be elevated in all PF-ILD subsets. The efficacy of Nintedanib in patients with other chronic non-IPF fibrosing ILDs with a progressive phenotype is being investigated in the INBUILD trial and the results are anticipated to be published in the autumn of 2019 [22]. We wanted to explore whether Nintedanib predominately targeted proteins (PDGF-AA, PDGF-BB, FGF-2, VEGF, and M-CSF) were elevated in whole lung homogenates from IPF, SSc-ILD and other PF-ILD compared to healthy donors. More importantly, we hypothesized there would be no difference in these protein concentrations across the PF-ILD subsets (IPF, SSc-ILD, and other PF-ILD). If our hypothesis is correct, we suspect that clinical trials using Nintedanib for PF-ILD with or without IPF or SSc-ILD will show a relative reduction in the annual rate of decline in FVC (mL/yr) of approximately 45–50%.

## 2 Materials and methods

### 2.1 PF-ILD study cohort

The research protocol was ethically approved by the institutional Review Board in Los Angeles, CA, USA, (#16-000117) and all participants gave written informed consent for collection of lung homogenates and analyses. PF-ILD was diagnosed based on meeting at least 1 of the following criteria for ILD progression during 24 months prior transplant: (i) relative decline in FVC ≥10% predicted; (ii) relative decline in FVC ≥5–<10% predicted and increased extent of fibrotic changes on high resolution computed tomography (HRCT); (iii) relative decline in FVC ≥5–<10% predicted and worsened respiratory symptoms; (iiii) worsened respiratory symptoms and increased extent of fibrotic changes on HRCT [22]; (iiiii) listed for lung transplant due to an ILD. The study cohort consisted of patients with PF-ILD transplanted at University of California Los Angeles (UCLA) (*n* = 130) who underwent lung transplantation between 2005 and 2011 due to ILD. Inclusion criteria were (i) the presence of PF-ILD regardless of diagnosis or prior therapy; and (ii) explant lung tissue samples available for protein analyses. All lung material was collected at time of lung transplantation from the left native lung in the case of a bilateral lung transplant operation, or either the left or right native lung depending on the side of the single lung transplant. The explanted lung was sampled at the interface between severely fibrotic lung and less involved lung tissue. The control group consisted of 200 biopsies from normal donor lungs being used for lung transplantation. Donor biopsies were done after explant and prior to implantation in the recipient.

### 2.2 Clinical assessment of PF-ILD patients

The clinical diagnoses were determined by histological patterns of the explanted lung and from clinical impressions that were confirmed by chart review [1]. Clinical and demographic data, including lung function tests were available for all included cases. Further changes in lung function were evaluated from time at listing for lung transplant to the time point of lung transplantation. Total FVC decline and annual FVC decline (total FVC decline divided by the time from listing to transplant) was assessed. Changes in FVC (% predicted) were defined as follows: FVC improvement (FVC increase of ≥5%), stable FVC (FVC decline or improvement of <5%), moderate decline (FVC decline of 5–10%), and significant decline (FVC decline of >10%) [23], [24], [25], [26]. The ILD- Global Alignment and Proportion (GAP) score was calculated [27].

### 2.3 Sample processing and protein measurement

Explanted ILD and donor lung samples were immediately placed in cold media and then on ice and transferred to the laboratory where they were homogenized and sonicated in anti-protease buffer using a method as previously described [28]. Specimens were centrifuged at 900 g for 15 min, filtered through a 1·2 μm sterile Acrodiscs (Gelman Sciences, Ann Arbor, MI [27]), and frozen at −70 °C until thawed for protein quantification analyses. The cytokines, growth factors and anigiogenic protiens were determined by bead based multiplex assays. Specifically, the concentrations of PDGF-AA, PDGF-BB, FGF-2, VEGF (VEGF-A) and M-CSF were all assayed via Luminex according to manufacturer instructions (Millipore, Billerica MA).

### 2.4 Statistical analyses

Analyses were performed by SPSS version 25 by AMHV and JB. Pearson Chi-square test, Fisher's exact test and Kruskal–Wallis *t*-test were used as appropriate. One-way analysis of variance (ANOVA) was used to compare means across variables with more than two groups. Pearson and Spearman coefficients were applied for correlation analyses of continuous variables as appropriate.

## 3 Results

### 3.1 Study cohort demographic and clinical characteristics at the time of lung transplant

**Fig. 1:**
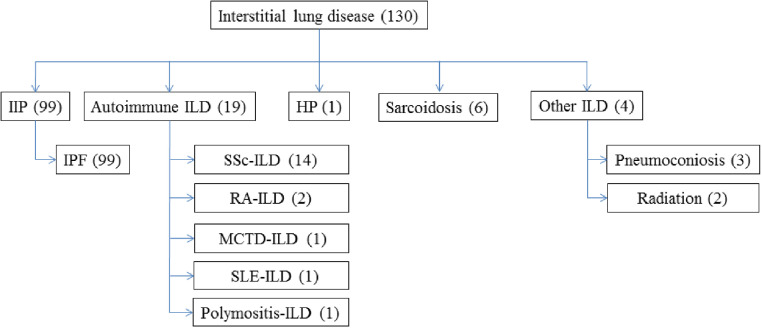
Types of interstitial lung disease (ILD) with progressive fibrosis ILD included in the study. ILD: interstitial lung disease; IIP: Idiopathic interstitial pneumonia; IPF: idiopathic pulmonary fibrosis; SSc: systemic sclerosis; RA: rheumatoid arthritis; MCTD: mixed connective tissue disease; SLE: systemic lupus erythematosus; HP: hypersensitivity pneumonitis.

The study cohort consisted of 130 PF-ILD cases, of which 99 (76%) had IPF, 14 (11%) had SSc-ILD and 17 (13%) other forms of ILD, as shown in Fig. 1. The total cohort median age was 59 years (SD 10·1), 29% were females, 45% were Caucasian, 12% Black, and 35% Hispanic (Table 1). Other clinical and demographics are shown in Table 1. None of the included PF-ILD patients were on anti-fibrotic treatment with Nintedanib or Pirfenidone at the time of lung transplantation. Of the 31 non-IPF PF-ILD patients, 9 (29%) were ever treated with Mycophenolate Mofetil, 7 (23%) with Cyclophosphamide, 7 (23%) with Prednisone monotherapy, 5 (16%) with Azathioprine, 3 (10%) with Hydroxychloroquine, 2 (6%) with Methotrexate, and 5 (16%) had been without immune-modulating treatment. In total, 14 (45%) had a combination of prednisone with a prednisone sparing agent such as Mycophenolate Mofetil, Cyclophosphamide, Azathioprine, Hydroxychloroquine or Methotrexate.

**Table 1:** Clinical characteristics and demographics of all included cases with progressive fibrosing interstitial lung disease (ILD) and segregated by idiopathic pulmonary fibrosis (IPF), systemic sclerosis associated ILD (SSc-ILD) and other ILD.

Clinical characteristics	All PF-ILDs (*n* = 130)	IPF (*n* = 99)	SSc-ILD (*n* = 14)	Other PF-ILD (*n* = 17)
Female gender, *n* (%)	38 (29)	25 (25)	7 (50)	6 (35)
Age at transplantation, years (SD)	59 (10.1)	64 (5·1)	49 (10·4)	49 (9·2)
BMI, (SD)	26 (4·0)	26 (3·9)	26 (4·3)	24 (4·6)
Race, *n* (%)				
White	58 (45)	43 (43)	8 (57)	7 (41)
Black	15 (12)	10 (10)	2 (14)	3 (18)
Asian	3 (2)	2 (2)	1 (7)	0 (0)
Hispanic	46 (35)	38 (38)	2 (14)	6 (35)
Other	8 (6)	5 (5)	1 (7)	1 (6)
Time on waiting list, months (SD)"	4·4 (5·6)	3·6 (4·6)	9·0 (10·8)	5·8 (4·4)
FVC, % (SD)				
At listing	43 (14·5)	44 (15·1)	43 (9·7)	39 (13·3)
At Transplant	42 (13·9)	42 (14·3)	32 (11·8)	36 (12·9)
Total FVC decline, % (SD)	−1·3 (6·4)	−1·3 (6·1)	−0·6 (7·2)	−2·2 (7·8)
Annual FVC decline, % (SD)	−7·1 (23·1)	−7·7 (23·6)	−0·1 (0·6)	−0·2 (0·65)
Moderate FVC decline, *n* (%)	20 (15)	15 (15)	2 (14)	3 (18)
Significant FVC decline, *n* (%)	10 (8)	7 (7)	1 (7)	2 (12)
DLCO, % (SD)				
At Transplant	29 (11·6)	29 (9·6)	22 (11·9)	34 (20·2)
Functional assessment				
6 MWD, *m* (SD)	110 (128)	96 (112)	101 (128)	186 (188)
ILD GAP score, *n* (%))				
0–1	7 (5·4)	0 (0)	2 (14·3)	5 (29·4)
2–3	23 (17·7)	3 (3·0)	11 (78·6)	9 (52·9)
4–5	50 (38·5)	47 (47·5)	0 (0)	3 (17·6)
>5	50 (38·5)	49 (49·5)	1 (7·1)	0 (0)

ID: interstitial lung disease; PF-ILD: progressive fibrosing ILD; SSc: systemic sclerosis; No: number; SD: standard deviation; BMI: Body mass index; FVC: forced vital capacity; 6MWD: six-minute-walking-distance; m: meters; GAP: Gender, Age, and Physiology index.

All included subjects had by definition PF-ILD. From the time of listing to the day of lung transplantation, 6 (5%) showed FVC improvement, 96 (74%) stable lung function and 28 (22%) further progression of their ILD. The PF-ILD group as a whole, at time of listing for lung transplantation had a mean diffusing capacity for carbon monoxide (DLCO) of 22% and mean FVC of 43%. A mean 4.4 months (SD 5·6) later at time of lung transplant the mean FVC was 42% (Table 1). The ILD-GAP score index is shown in Table 1.

### 3.2 PDGF, FGF, VEGF and M-CSF concentrations are increased in lung homogenates from patients with progressive fibrosing ILD compared to healthy lung donors

**Fig. 2:**
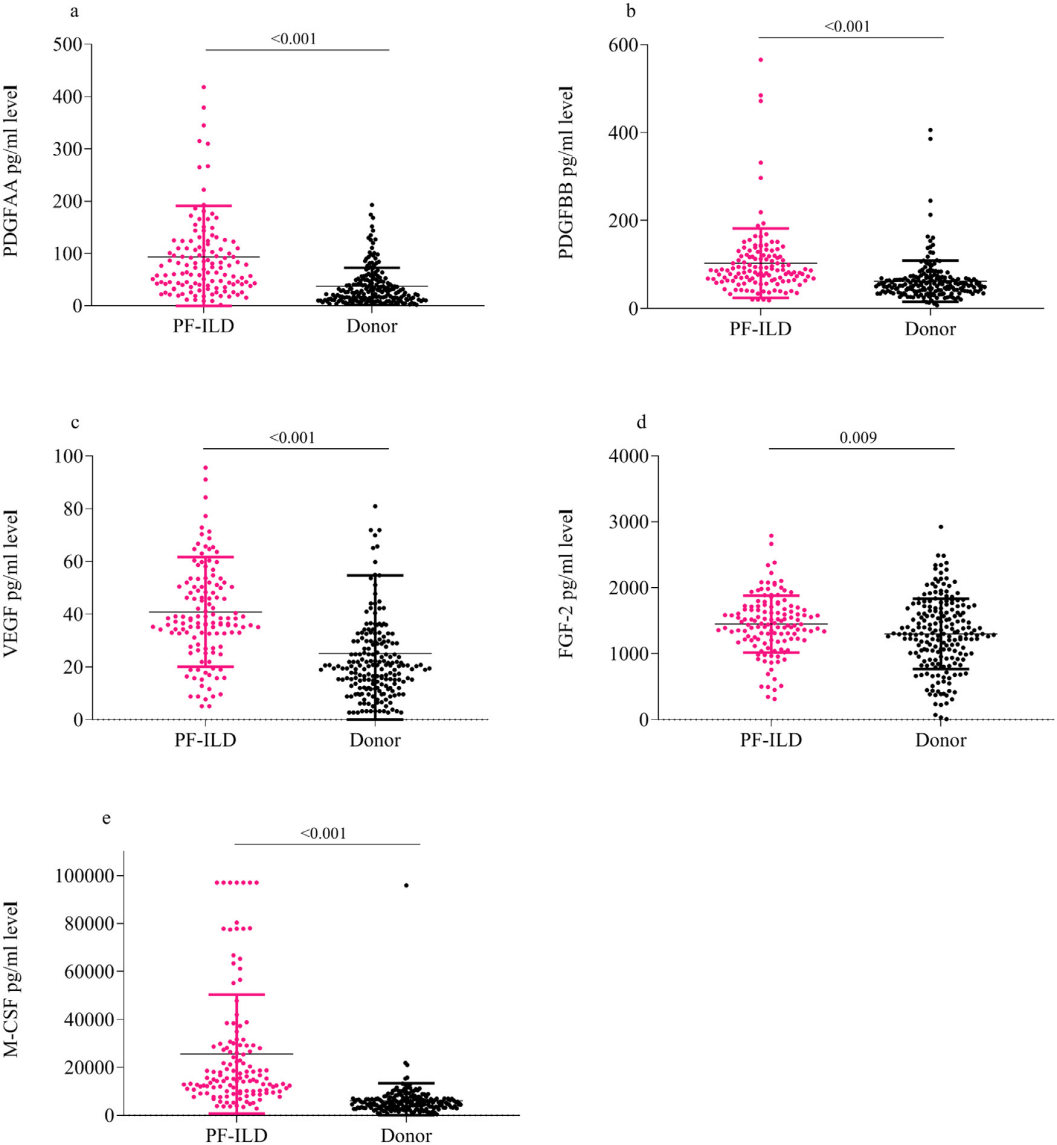
Augmented mean (SD) PDGF-AA, PDGF-BB, FGF-2, VEGF and M-CSF concentrations in PF-ILD lung homogenates [measured by *t*-test]. (a–d) PDGF-AA, PDGF-BB, VEGF, FGF-2 and M-CSF protein concentration in fresh explanted lung tissue homogenates from the UCLA PF-ILD cohort (*n* = 130), as compared to healthy lung tissue homogenate controls from donor lungs (*n* = 200).

**Fig. 3:**
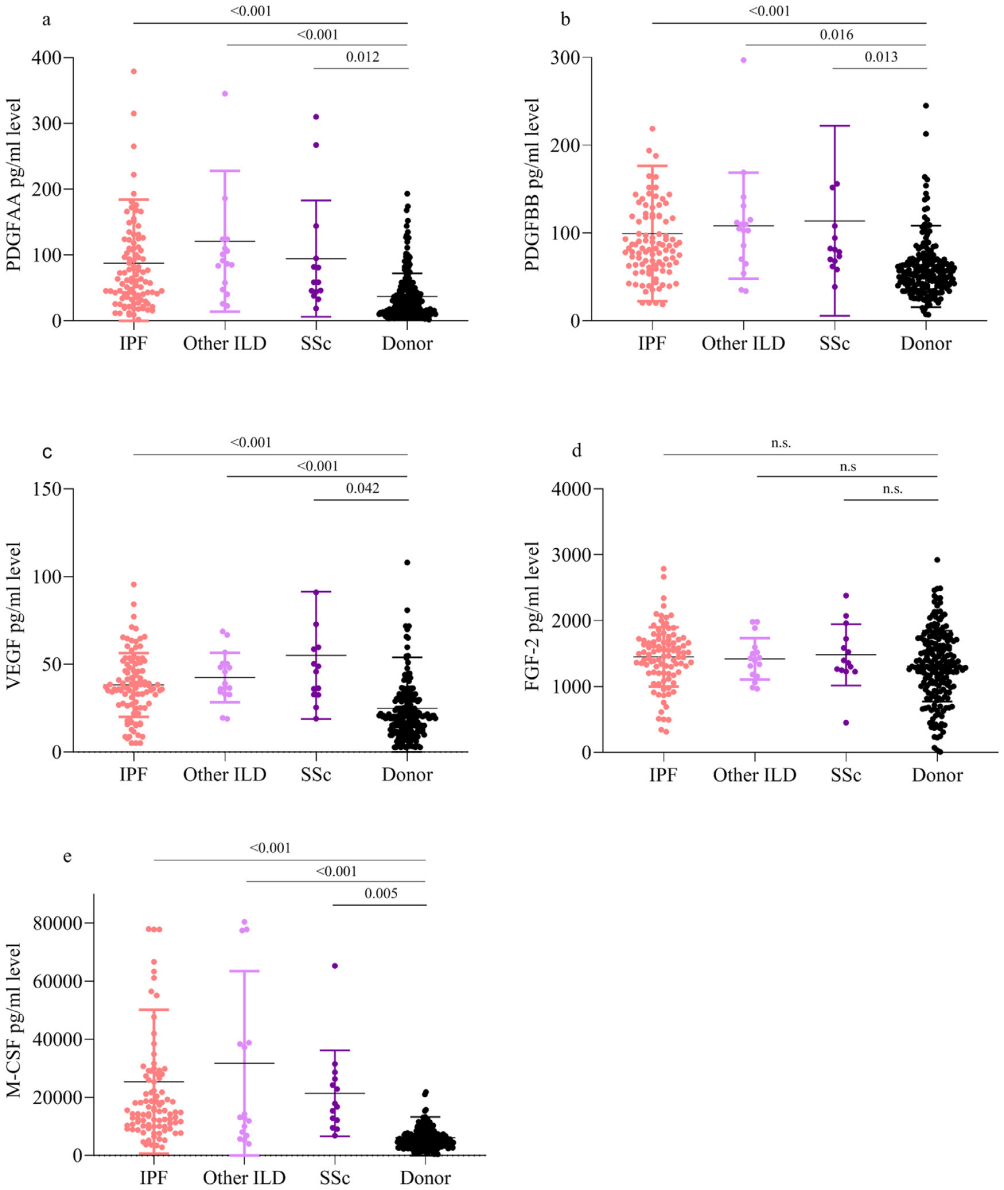
Augmented mean (SD) PDGF-AA, PDGF-BB, FGF-2, VEGF and M-CSF concentrations in PF-ILD lung homogenates segregated by the underlying disease [measured by one-way analysis of variance (ANOVA)]. (a–e) PDGF-AA, PDGF-BB, VEGF, FGF-2 and M-CSF protein concentration in explanted lung tissue homogenates from the UCLA ILD cohort (*n* = 130) segregated by IPF (*n* = 99), SSc-ILD (*n* = 14) and other ILDs (*n* = 17), as compared to healthy lung tissue homogenate controls from donor lungs (*n* = 200).

In whole lung homogenates, PDGF-AA, PDGF-BB, FGF-2, VEGF and M-CSF mean concentrations were significantly increased in the PF-ILD lungs (*n* = 130) compared to healthy lung donors (*n* = 200) (PDGF-AA 93·0pg/ml [±97·2] vs. 37·5 pg/ml [±35·4], *p* < 0·001; PDGF-BB 102·5 pg/ml [±78·8] vs. 61·9 pg/ml [±47·0], *p* < 0·001; FGF-2 1442·4pg/ml [±426·6] vs. 1201·7 pg/ml [±535·2], *p* = 0·009; VEGF 40·6 pg/ml [±20·1] vs. 24·9 pg/ml [±29·5], *p* < 0·001; and M-CSF 25526 pg/ml [±24,799] vs. 6120 pg/ml [±7245], *p* < 0·001) as shown in Fig. 2. When segregated by ILD aetiology (IPF, SSc-ILD, and other ILD), the mean protein concentrations of PDGF-AA, PDGF-BB, VEGF and M-CSF; but not FGF-2 were elevated with each category of PF-ILD as compared to healthy controls measured by ANOVA (Fig. 3(a)–(e)).

### 3.3 PDGF, FGF, VEGF and M-CSF concentrations are not different in lung homogenates from patients with progressive fibrosing ILD due to IPF, SSC-ILD or other ILDs

**Fig. 4:**
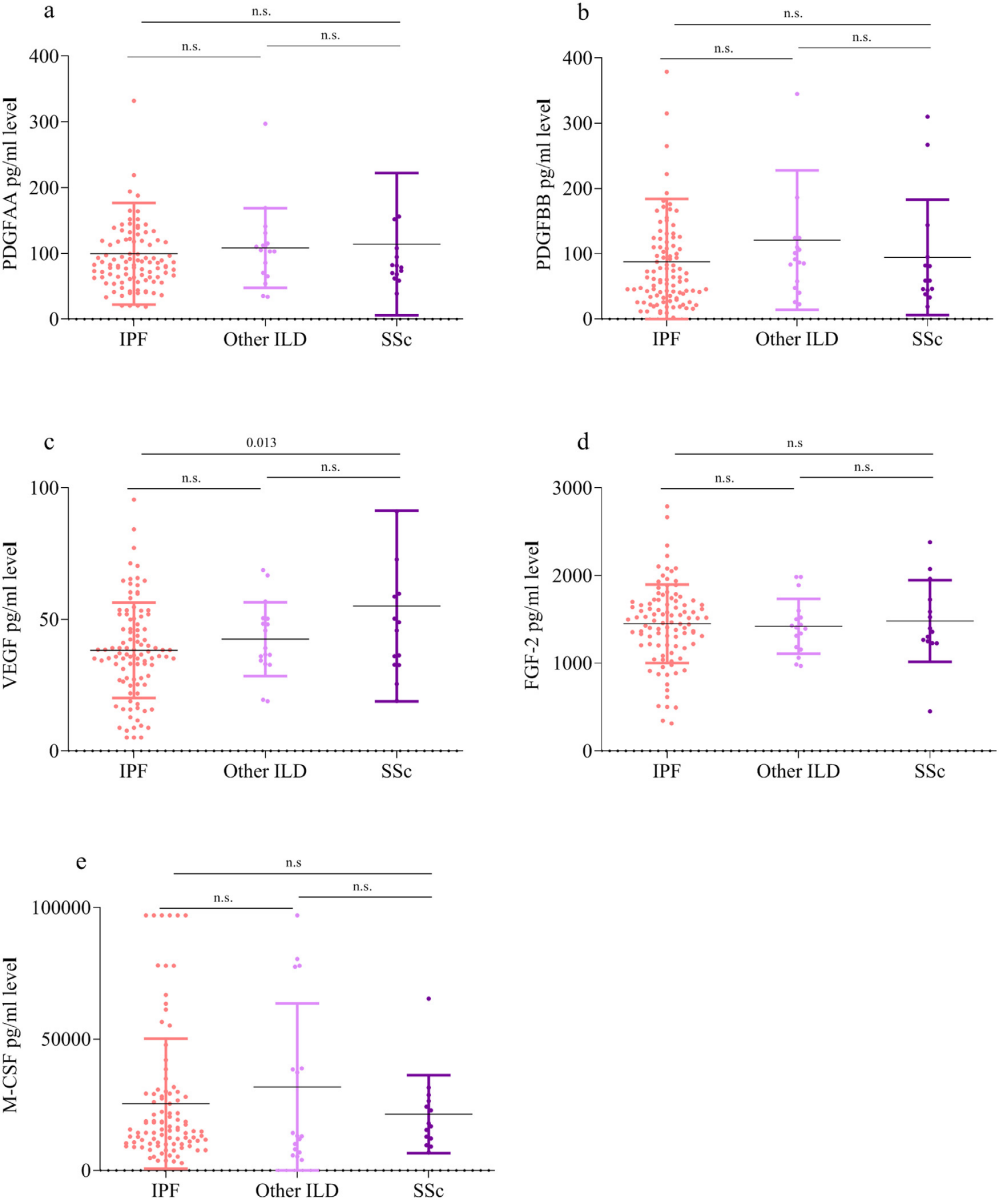
Similar augmented mean (SD) concentrations of PDGF-AA, PDGF-BB, FGF-2, VEGF and M-CSF (SD) in PF-ILD lung homogenates segregated by the underlying disease [measured by one-way analysis of variance (ANOVA)]. (a–e) PDGF-AA, PDGF-BB, VEGF, FGF-2 and M-CSF protein concentration in explanted lung tissue homogenates from the UCLA ILD cohort (*n* = 130) segregated by IPF (*n* = 99), SSc-ILD (*n* = 14) and other ILDs (*n* = 17).

In whole lung homogenates, mean protein concentrations of PDGF-AA, PDGF-BB, FGF-2, VEGF and M-CSF did not show significant differences between the underlying ILD aetiologies (IPF, SSc-ILD and other ILD), except for VEGF, which was significantly higher in SSc-ILD compared to IPF measured by ANOVA (Fig. 4(a)–(e)). These data represent PF-ILD predominately due to the need and receiving a lung transplant for ILD. We also performed a subset analysis that used a more strict criteria of PF-ILD based on moderate or severe FVC decline (≥ 5%) [22]. In this analysis mean protein concentrations of PDGF-AA, PDGF-BB, FGF-2, VEGF and M-CSF did not show significant differences between the underlying ILD aetiologies (IPF, SSc-ILD and other ILD) using ANOVA**.** Furthermore, protein concentrations of PDGF-AA, PDGF-BB, FGF-2, VEGF and M-CSF did not show correlations with demographic (age, sex, race), and baseline lung function characteristics (FVC%, DCLO%, six minute walking distance (6MWD) and ILD-GAP index) between the underlying ILD aetiologies (IPF, SSc-ILD and other ILD) with the exception of a weak correlation of age and FGF-2 (0·37, *p* < 0·001) for IPF, a correlation of age and PDGF-BB in SSc-ILD (0·59, *p* = 0·027) and correlations between 6MWD and FGF-2 in other PF-ILD (0·57, *p* = 0.036).

## 4 Discussion

Although the subsets of ILD may differ with regard to their initiation/trigger (e.g., unknown for IPF and sarcoidosis, autoantigen with adaptive immune responses for CTD-ILD, antigen exposure for HP, radiation for x-ray induced fibrosis, coal or silicosis or siderosis for pneumoconiosis); many patients develop progressive fibrotic changes (e.g., PF-ILD) with similar clinical courses regardless of ILD aetiology [7,20]. Therefore, it is possible that despite multiple different aetiologies of ILD, the relentless progression of the ILD may have common molecular pathways involved [7]. Importantly, this line of thought led to the concept of trying drugs approved for slowing the progression of IPF to be explored in other ILD [6,21]. Nintedanib, a tyrosine kinase inhibitor targeting the PDGF, FGF, VEGF and M-CSF pathways, has shown comparable efficacy in IPF and SSc-ILD [5,^16^,21]. We hypothesized that Nintedanib targeted molecular pathways (PDGF, FGF, VEGF and M-CSF) would be augmented to a similar degree across PF-ILD regardless of aetiology. In this study, we confirmed that these pathways were augmented to a similar magnitude in all PF-ILD lung tissue as compared to controls, suggesting that Nintedanib treatment should have comparable efficacies across all PF-ILD regardless of aetiology.

Nintedanib is an intracellular inhibitor of tyrosine kinase receptors that is predominately involved in PDGFRα, PDGFRβ, FGFR 1–3 and VEGFR 1–3 pathways [16]. In vitro and in vivo studies have suggested that all of these pathways are involved in ILD pathobiology via inflammation/cell activation, fibroplasia and vascular remodelling [15,16]. In addition, Nintedanib also inhibits CSF1R (colony stimulating factor 1 receptor) and to some degree limits the Src family kinase Lck (lymphocyte-specific tyrosine protein kinase) as well as 20 other kinases [16]. To date, it has not been investigated whether the predominant pathways Nintedanib inhibits are collectively augmented in the lungs of patients with PF-ILD. We set out to determine whether the concentrations of Nintedanib-specific targeted proteins are elevated in explanted lung tissues from patients with PF-ILD, regardless of aetiology. More specifically, we determined the concentrations of PDGF-AA, PDGF-BB, FGF-2, VEGF and M-CSF in whole lung homogenates from patients with PF-ILD of different aetiologies including IPF, SSc-ILD, other CTD-ILD, sarcoidosis and exposure related-ILD, and compared them to normal lung tissue. All subgroups of PF-ILD had augmented levels of PDGF-AA, PDGF-BB, VEGF and M-CSF as compared to healthy donor lungs. These findings are consistent with the pathobiology of ongoing fibrosis with PDGF being involved in fibroblast proliferation, invasion and matrix deposition [11]. Similarly, VEGF and FGF-2 also have been shown to be involved in fibroproliferation via effects on leukocytes and well as angiogenic activities [12,14]. However, the role of angiogenesis in IPF and PF-ILD is controversial. For instance, some studies demonstrate increased and others reduced vascular density in the IPF lung, which likely has to do with the heterogeneity of pathology of this disease [29,30]. Similarly, there is an ongoing debate as to whether or not angiogenic or angiostatic mediators in conjunction with altered endothelial cells may be protective or harmful in supporting interstitial fibrosis, epithelial cell repair, and pulmonary hypertension associated ILD during the pathobiology of pulmonary fibrosis [31], [32], [33], [34]. M-CSF is important in the survival and the recruitment of mononuclear phagocytes that are phenotypically distinct and profibrotic, suggesting its role in fibroplasisa [35]. Thus, it makes sense that these pathways would, in part, be involved in PF-ILD patients with ongoing loss of lung function.

For the most part, we did not find significant differences in concentration of the growth factors/ angiogenic molecules/ cytokines between the different PF-ILD subsets (IPF, SSc-ILD and other PF-ILD). Additionally, we did not find that any of these protein concentrations associated with type of PF-ILD, FVC% and ILD-GAP index at the time of transplant or severity of PF-ILD using FVC% decline. These findings make it tempting to support the notion of “lumping” the different PF-ILD into one entity with regards to anti-fibrotic treatment, rather than identifying specific subgroups. However, there are many other molecular pathways involved in pulmonary fibrosis development and further progression. For instance Th2 and Tfh immune responses, connective tissue growth factor or other growth factors, and TGF-β may be involved in specific diseases and not in all, but were not tested in this study [13,^14^,^36^,37]. Importantly, this is why funding needs to continue to focus on the reductionist (determining the specific contribution of individual pathways during PF-ILD) and non-reductionists (evaluating multiple pathways together found by the reductionist during PF-ILD) approaches to PF-ILD, which when combined will have the greatest clinical impact. Collectively, these methodologies are likely to produce novel drugs that will stabilize or improve lung function for patients with PF-ILD. Moreover, determining the specific endotypes of PF-ILD may be the most advantageous way to determine individual treatments in a more personalized medical approach, which should limit adverse events as well as further improve long-term outcomes/survival.

As with all studies, there are limitations. In this study, we evaluated all end-stage PF-ILD collected at the time of lung transplantation. Thus, these samples involve lung tissues with severe fibrosis, which may or may not extrapolate to more mild and moderate PF-ILD. We evaluated whole lung homogenates and quantitated mediators from all cell types in bulk as compared to the expression from specific cell types such as epithelial cells, endothelial cells, fibroblasts and leukocyte subpopulations. We did not have information available as to whether or not the IPF patients were on immunomodulatory therapies or had pulmonary hypertension, which may or may not affect the mediators we quantitated.

In conclusion, we have found that Nintedanib target pathways (PDGF-AA, PDGF-BB, FGF-2, VEGF and M-CSF) are augmented in all PF-ILD subsets and importantly their concentrations are similar across PF-ILD subsets suggesting that Nintedanib treatment should have similar efficacy in PF-ILD regardless of aetiology (e.g., IPF, SSc-ILD and other PF-ILD). We speculate that clinical trials using Nintedanib for PF-ILD with or without IPF or SSc-ILD should show a similar relative reduction in FVC decline as seen in IPF and SSc-ILD (∼45–50%) based on these data.

## Data sharing

Not available.

## Declaration of Competing Interest

AMHV has received research funding and/or consulting fees or other remuneration from Actelion, Boehringer Ingelheim and Roche.

SSW and JAB have received research funding and/or consulting fees or other remuneration from Boehringer Ingelheim and Genentech.

EV has received research funding and/or consulting fees or other remuneration from Boehringer Ingelheim.

JAB has received research funding and/or consulting fees or other remuneration from Boehringer Ingelheim and Genentech.
